# Transport lattice models of heat transport in skin with spatially heterogeneous, temperature-dependent perfusion

**DOI:** 10.1186/1475-925X-3-42

**Published:** 2004-11-17

**Authors:** TR Gowrishankar, Donald A Stewart, Gregory T Martin, James C Weaver

**Affiliations:** 1 Harvard-MIT Division of Health Sciences and Technology, Massachusetts Institute of Technology, Cambridge, MA 02139, USA; 2 Thermal Technologies, Inc., Cambridge, MA 02139, USA

## Abstract

**Background:**

Investigation of bioheat transfer problems requires the evaluation of temporal and spatial distributions of temperature. This class of problems has been traditionally addressed using the Pennes bioheat equation. Transport of heat by conduction, and by temperature-dependent, spatially heterogeneous blood perfusion is modeled here using a transport lattice approach.

**Methods:**

We represent heat transport processes by using a lattice that represents the Pennes bioheat equation in perfused tissues, and diffusion in nonperfused regions. The three layer skin model has a nonperfused viable epidermis, and deeper regions of dermis and subcutaneous tissue with perfusion that is constant or temperature-dependent. Two cases are considered: (1) surface contact heating and (2) spatially distributed heating. The model is relevant to the prediction of the transient and steady state temperature rise for different methods of power deposition within the skin. Accumulated thermal damage is estimated by using an Arrhenius type rate equation at locations where viable tissue temperature exceeds 42°C. Prediction of spatial temperature distributions is also illustrated with a two-dimensional model of skin created from a histological image.

**Results:**

The transport lattice approach was validated by comparison with an analytical solution for a slab with homogeneous thermal properties and spatially distributed uniform sink held at constant temperatures at the ends. For typical transcutaneous blood gas sensing conditions the estimated damage is small, even with prolonged skin contact to a 45°C surface. Spatial heterogeneity in skin thermal properties leads to a non-uniform temperature distribution during a 10 GHz electromagnetic field exposure. A realistic two-dimensional model of the skin shows that tissue heterogeneity does not lead to a significant local temperature increase when heated by a hot wire tip.

**Conclusions:**

The heat transport system model of the skin was solved by exploiting the mathematical analogy between local thermal models and local electrical (charge transport) models, thereby allowing robust, circuit simulation software to obtain solutions to Kirchhoff's laws for the system model. Transport lattices allow systematic introduction of realistic geometry and spatially heterogeneous heat transport mechanisms. Local representations for both simple, passive functions and more complex local models can be easily and intuitively included into the system model of a tissue.

## Background

Heat transfer in biological systems is relevant in many diagnostic and therapeutic applications that involve changes in temperature. For example, in hyperthermia the tissue temperature is elevated to 42–43°C using microwave [1,2], ultrasound [3], or laser light [4]. There has also been long standing interest in thermal properties of skin [5] in order to understand conditions leading to thermal damage (burns) to skin, usually involving contact of skin to hot objects [6], in which local thermal conduction and heat capacity are dominant. Investigation of such bioheat transfer problems requires the evaluation of temporal and spatial distributions of temperature. This class of problems has been traditionally addressed using the Pennes bioheat equation [7,8]. Here we show that a transport lattice approach [9] can solve bioheat problems. This method is illustrated by solving models for skin contact heating used in transcutaneous blood gas monitoring and for spatially distributed heating due to 10 GHz microwave radiation.

Contact heating is used in transcutaneous blood gas monitoring, in which oxygen is transported out of the vasodilated capillary bed to a surface mounted oxygen sensor. Heating is used to achieve vasodilation. In 1851 it was already known that "skin breathing" occurs, in which oxygen diffuses out of ambient air into the body, supplying of order 1% of the body's oxygen uptake [10]. Typically the ambient air temperature even with clothing insulation causes the skin surface temperature to be significantly cooler than body core temperature. Much later, in 1957, it was shown that elevated skin temperature caused an outward diffusive flux of oxygen, so that oxygen could be measured at the surface of the skin [11]. The basic idea is that contact surface heating results in heat transport into the body, such that the outer portion of the dermis (the site of the outermost blood capillaries) experiences a significant increase in perfusion. This temperature-dependent perfusion "arterializes" the blood content of the capillaries, such that the oxygen concentration is closer to that of arterial blood, because to a good approximation capillary flow increases faster than oxygen transport out of the capillaries.

Initial clinical demonstration with neonates occurred in 1969 when a polarographic electrode placed on the head was used to measure oxygen partial pressure [12]. Since the early studies significant development has taken place [13-16]. A basic issue of safety is involved, as sensor contact is often prolonged (1–8 h) in which a heated sensor (typically 45°C) with contacting material of high thermal diffusivity is placed against the skin.

Spatially distributed heating of skin and deeper tissue by electromagnetic fields and ultrasound is also of established interest [17-20]. Microwave electromagnetic radiation is incident on tissue under a variety of exposure conditions. As an example, we consider 10 GHz microwave exposure. In this case, the penetration depth is approximately 3 mm so that most of the power is deposited within the outer region of the skin. Accurate prediction of the temperature distribution in skin exposed to microwave radiation is important in understanding both beneficial and harmful effects [21-23].

In hyperthermia, tissue is heated to enhance the effect of conventional radio- or chemotherapy. By delivering thermal energy, the tissue is stimulated to increase the blood flow by thermoregulation in order to remove the excess heat. The common method to produce local heating in the human body is the use of electromagnetic waves.

Many of the bioheat transfer problems have been modeled using the Pennes equation, which accounts for the ability of tissue to remove heat by both passive conduction (diffusion) and perfusion of tissue by blood. Perfusion is defined as the nonvectorial volumetric blood flow per tissue volume in a region that contains sufficient capillaries that an average flow description is considered reasonable. Most tissues, including much of the skin and brain, are highly perfused, with a perfusion coefficient denoted by *ω *(traditionally with units of 100 ml/100 g min = 1 ml g ^-1 ^min ^-1^). Alternatively, *ω *can be replaced by *ω*_m_, the nondirectional mass flow associated with perfusion. Perfusion is valid on the spatial scale of ~100 *μ*m. The contributions of heat conduction and perfusion are combined in the Pennes bioheat equation [7,8], which we use in a form [24] that employs *ω*_m _(SRI units of kgm^-3 ^s^-1^),



Here, *ρ*, *c*, *k *are the density, specific heat and thermal conductivity of tissue, respectively and *c*_*b*_, is the specific heat of blood, *ρ*_*b *_is the density of blood, *T *is local tissue temperature, *T*_a _is a reference temperature (arterial blood), *t *is time, *Q*_m _is the metabolic heat production per volume, and *P*(*z*, *t*) is the heat deposited per volume due to spatially distributed heating. In this general form, *ω*_*m *_is a function of temperature to include the specific case of temperature dependent perfusion. Vascularized tissue generally experiences increased perfusion as temperature increases [25,26]. Because of thermoregulation skin blood flow rises 15 fold to 100 ml l00 g min^-1^, often with a time lag of minutes.

Prediction of heat transport has long been carried out by both analytical and numerical methods [27,28]. The temperature rise for constant (temperature independent) perfusion has been predicted by traditional analytical methods based on Eq. 1, which can be solved analytically for simple geometries [22,29] or by finite element models for more realistic, complicated tissue geometry [30-32]. Models which include temperature-dependent increases in perfusion are more difficult to solve, but the case of a linear temperature dependence have been described using analytical expressions [33] and numerical simulations [24]. The bioheat transfer (Eq. 1) has been used in a wide range of applications to describe heat transport in blood perfused tissues, [34] and solved by a variety of methods. An adaptive finite element method was used to optimize the nonlinear bioheat equation for optimizing regional hyperthermia [35]. Two-dimensional biothermal models of ultrasound applicators based on the bioheat equation were solved by finite difference equation [36]. The boundary element and finite difference methods have also been used to solve the bioheat equation [37-41]. Recently, closed form analytical solutions to the bioheat transport problems with space and transient heating were reported using Green's function method [42].

Here we show that the transport lattice approach can be used to model transport of heat by conduction and temperature-dependent blood perfusion. This method employs a network of locally interacting transport, storage and source models that are solved as a system model by Kirchhoff's laws. Although Kirchhoff's laws can be used to describe transport of heat (and of molecules), usually charge transport is treated. Indeed, there is an extensive literature and robust methodology for solving large electric circuits [43]. For this reason, we use electrical circuits which provide mathematical analogs to heat transport and storage. Transport lattices allow systematic introduction of realistic geometry and spatially heterogeneous heat transport mechanisms. One attribute of a transport lattice model is that local representations for both simple, passive functions (e.g. heat storage via fixed heat capacitance and thermal conduction via fixed thermal conductivity) and more complex local models (e.g. nonlinear, temperature-dependent perfusion and spatially non-uniform perfusion in which the time lag of perfusion onset can be selected) can be easily and intuitively included into the system model of a tissue. It is a fundamentally modular approach in which local models can be introduced or removed.

## Methods

**Figure 1:**
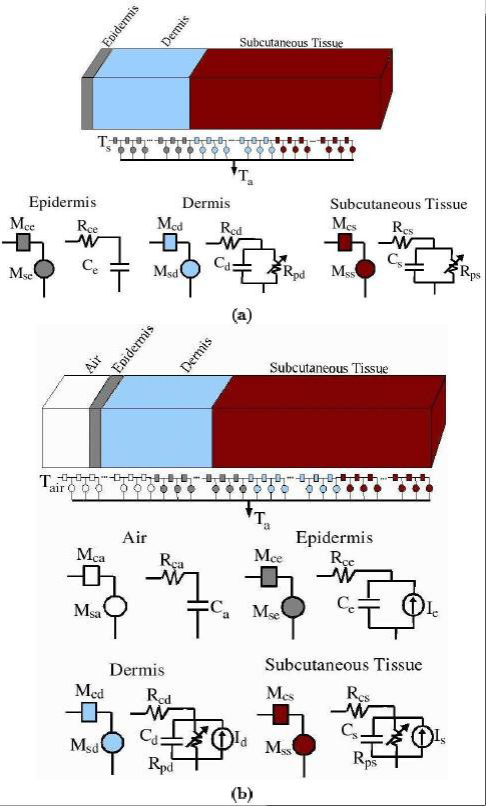
**Transport lattice method – Geometry with transport, source and storage models**. The one-dimensional model of human skin (top) is represented by a lattice of conduction models (*M*_*c*_) and source, storage and sink models (*M*_*s*_). The subscripts denote the different layers of the model, namely, a: air, e: epidermis, d: dermis and s: subcutaneous tissue. The equivalent circuit models are shown for each layer: *R*_*c *_represents heat conduction, *R*_*p *_represents heat removal by perfusion (not present in epidermis) and *C *represents heat storage. The conduction model, *M*_*c*_, is represented by *R*_*c *_while the storage, perfusion and power input model, *M*_*s*_, is represented by the combination of *R*_*p*_, *C *and *I *(Table 2). **(a) Surface Contact Heating**: The surface temperature *T*_*s *_is elevated from 33°C to 45°C at t = 0. **(b) Spatially Distributed Heating**: A layer of air contacting the skin is added to the model with the air temperature (*T*_*air*_) held at 25°C. The local microwave power dissipation is represented by the current source (I) at each node. The arterial reference temperature *T*_*a *_is represented by a common node. A ladder-like network of variable resistors, *R*_*p*_, represents the temperature dependent perfusion in dermis and subcutaneous tissue.

Here we extend the transport lattice modeling approach previously demonstrated for electrical fields and currents in single and multiple cells [9] and supra-electroporation of cells by submicrosecond pulses [44] to describe heat transport within a multilayered skin model. A related approach has been described for analysis of calorimeters to measure specific heat of liquids [45]. Circuit analysis has long been used to solve problems that can be described by differential equations [45-49]. Here we use a modular approach in which the skin is represented by three layers, each with many interconnected local models that account for the local heat storage (heat capacity) and local transport by both conduction and perfusion (Fig. 1). The different parameters employed in the model and their values are listed in Table 1. We model two cases of skin heating: surface contact heating and spatially distributed heating.

**Table 1:** Thermal and electrical property values assigned to different layers of skin.

Air			
*t*_*a*_	thickness	500 *μm*	
*N*_*a*_	number of lattice elements	100	
ℓ	lattice node spacing	5 *μm*	
*k*_*a*_	thermal conductivity	0.0263 *Wm*^-1 ^°*C*^-1^	
*ρ*_*a*_	density	1.3 *kg m*^-3^	
*c*_*a*_	specific heat	1004 *Jkg*^-1 ^°*C*^-1^	

Epidermis			

*t*_*e*_	thickness	80 *μm*	[52]
*N*_*e*_	lattice elements	80	
ℓ	lattice node spacing	1 *μm*	
*σ*_*e*_	electrical conductivity	8.01 *Sm*^-1^	[70]
*ε*_*e*_	relative permittivity	31.3	[70]
*η*_*e*_	penetration depth	3.8 mm	[70]
*λ*_*e*_	wavelength	5.2 mm	[70]
*k*_*e*_	thermal conductivity	0.23 *Wm*^-1 ^°*C*^-1^	[52]
*ρ*_*e*_	density	1200 *kg m*^-3^	[52]
*c*_*e*_	specific heat	3590 *Jkg*^-1 ^°*C*^-1^	[52]
*ω*_*e*_	perfusion rate	0 *m*^3^*s*^-1^*m*^-3 ^tissue	[52]

Dermis			

*t*_*d*_	thickness	2000 *μm*	[52]
*N*_*d*_	lattice elements	100	
ℓ	lattice node spacing	20 *μm*	
*σ*_*d*_	electrical conductivity	8.01 *Sm*^-1^	[70]
*ε*_*d*_	relative permittivity	31.3	[70]
*η*_*d*_	penetration depth	3.8 mm	[70]
*λ*_*d*_	wavelength	5.2 mm	[70]
*k*_*d*_	thermal conductivity	0.45 *Wm*^-1 ^°*C*^-1^	[52]
*ρ*_*d*_	density	1200 *kg m*^-3^	[52]
*c*_*d*_	specific heat	3300 *Jkg*^-1 ^°*C*^-1^	[52]
*ω*_*d*_	perfusion rate	1.25 × l0^-3 ^*m*^3^*s*^-1^*m*^-3 ^tissue	[52]

Subcutaneous Tissue			

*t*_*s*_	thickness	18000 *μm*	[52]
*N*_*s*_	lattice elements	100	
ℓ	lattice node spacing	180 *μm*	
*σ*_*f*_	electrical conductivity	0.585 *Sm*^-1^	[70]
*ε*_*f*_	relative permittivity	4.60	[70]
*η*_*f*_	penetration depth	19.6 mm	[70]
*λ*_*f*_	wavelength	13.9 mm	[70]
*k*_*s*_	thermal conductivity	0.19 *Wm*^-1 ^°*C*^-1^	[52]
*ρ*_*s*_	density	1000 *kg m*^-3^	[52]
*c*_*s*_	specific heat	2675 *Jkg*^-1 ^°*C*^-1^	[52]
*ω*_*s*_	perfusion rate	1.25 × 10^-3 ^*m*^3^*s*^-1^*m*^-3 ^tissue	[52]

Blood			

*c*_*b*_	specific heat	3770 *Jkg*^-1 ^°*C*^-1^	[52]
*ρ*_*b*_	density	1060 *kg m*^-3^	[52]

### Surface contact heating

The case of a fixed skin surface temperature is relevant to transcutaneous blood gas sensors, in which a skin-contacting sensor with controlled temperature, and a local source of heat of up to 45°C are employed to significantly increase perfusion within the outer capillary bed [50], thereby "arterializing" the capillary blood. This situation also represents heating at the skin surface by a heat source, or the skin contacting a hot object with a large thermal diffusivity, such as in thermal injury [51,52]. Surface heating may be either essentially constant (long duration) or transient (short duration). The latter is relevant to laser pulse application or flash skin burns. We model surface contact heating by considering step heating of skin surface to different temperatures at t = 0. The core temperature is assumed to be constant at the ambient temperature (T_a_). The boundary conditions are shown in Fig. 1.

### Spatially distributed heating

Spatially distributed heating occurs in skin exposed to penetrating, dissipative radiation such as microwave, ultrasound and laser light [51,53]. These heating methods often involve an exponentially decaying power transmission accompanied by reflection at the interface of regions with different electrical properties. We consider a uniform plane wave incident normally upon the skin surface, with a layer of air included to model the reflection at the skin/air interface. We also account for interference from reflections at the interface of dermis and subcutaneous fat and at the skin/air interface. The average absorbed power density, *P*(*z*, *t*), in epidermis and dermis (of thickness *d *= *t*_*e *_+ *t*_*d*_), in the range 0 <*z *≤ *d *is given by



where



and



and the average absorbed power density in the subcutaneous fat layer (*z *>*d*) is given by



where



where *P*(0, *t*) is the power density incident on the skin surface at time *t*, *E*(0, *t*) is the corresponding electric field amplitude, (*z*, *t*) is the propagating electric field in the epidermis and dermis, *E*(*d*, *t*) is the electric field at the dermis-subcutaneous fat interface, *η*_*d *_and *η*_*s *_are the penetration depths for dermis and subcutaneous fat, *λ*_*d *_and *λ*_*s *_are the wavelengths in dermis and subcutaneous fat, and *Z*_*a*_, *Z*_*d *_and *Z*_*s *_are the intrinsic impedances of air, dermis, and subcutaneous fat, respectively. Note that the incident power, *P*(0, *t*), is expressed as an area density whereas the absorbed power density, *P*(*z*, *t*), in the skin is expressed as a volume density. The summation of (*z*, *t*) in Eq. 2 was carried out to 10 terms, although only the first two terms are significant. The reflection (Γ) and transmission (*T*) coefficients at the skin/air (*sa*) and for skin/fat (*sf*) interfaces and the intrinsic impedances are given by [54]





where *σ*_*d *_and *σ*_*s *_are the conductivities of dermis and subcutaneous fat and *ε*_*d *_and *ε*_*s *_are the permittivities of dermis and fat, respectively (listed in Table 1), *μ*_0 _is the permeability of free space, *ε*_0 _us the permittivity of free space, and *f *= 10^10 ^Hz.

### Circuit model of heat conduction

A motivation for the transport lattice for heat conduction is the electrical equivalence of heat transport (a diffusion process [27]). We consider the well known equivalence of electrical and thermal conduction. Heat conduction is described using a thermal resistance, *R*, which relates the heat flow per unit time *Q *to the temperature difference Δ*T *as *Q *= (1/*R*)Δ*T*. In the case of heat conduction across a cube of thickness ℓ and area ℓ^2^, *R*_*c *_= ℓ/(*k*ℓ^2^) = (*k*ℓ)^-1 ^where *k *is the thermal conductivity of the slab material. Heat storage is described by the thermal capacitance, *C*, which for a slab is *C *= *ρc*_*p*_ℓ·ℓ^2 ^= *ρc*_*p*_ℓ^3 ^where *ρ *is the density of the slab material and *c*_*p *_is its specific heat. The associated thermal relaxation time is *τ*_*Q *_= *Q*/ = (*ρc*_*p*_ℓ^3^)/(*k*ℓ) = ℓ^2^/*α*, where *α *= *k*/(*ρc*_*p*_) is the thermal diffusivity. The heat conduction models for different layers of skin are shown in Fig. 1 as *R*_*c*_and *C *with subscripts identifying the particular skin layer.

### Circuit model of perfusion

Pennes bioheat equation provides an approximate description of heat transport by tissue conduction and by blood flow using a local temperature dependent conduction path to perfusing blood. This additional heat removal is proportional to the local temperature difference *T *- *T*_a_. Here, local heat removal by perfusion is described by a thermal resistor, *R*_*p *_= (ℓ^3^*ω*_*m*_*c*_*b*_*ρ*_*b*_), connected to a reference node at ambient temperature (Fig. 1) where *c*_*b *_is specific heat of blood, *ρ*_*b *_is the density of blood.

### Circuit model of surface heat loss

Unoccluded skin often transports heat across its outer surface via a combination of conduction into a boundary layer of air, convective movement of air, and black or gray body radiation. Because our emphasis here is on conduction and perfusion, we lump these surface transport mechanisms into a single heat transfer coefficient. The numerical value of this coefficient was determined by requiring the initial skin surface temperature to be *T*_s _= 33°C (before contacting the skin to a heated surface or applying microwave radiation). The surface heat loss for microwave heating is represented by a series of conduction models (*R*_*ca *_in Fig. 1). For contact heating the surface is initially set to 33°C and then raised to 45°C at t = 0.

### Circuit model of spatially distributed power deposition

Spatially distributed power deposition from 10 GHz radiation is modeled by representing Eqs. 2 and 3 by an equivalent local current (heat flux) source, *I*_*z *_= *P*(*z*, *t*)ℓ^3^, at each node (Fig. 1). That is, each node has a local power input based on Eqs. 2 and 3 multiplied by the local volume.

### Metabolic heat generation

Metabolic heat generation can also be represented by local sources, but these are set to zero in the present models. In a transport lattice model an additional heat source can be introduced at each node to represent heat generation by metabolism. Here, metabolism is assumed to maintain the baseline temperature at a constant value equal to the arterial blood temperature. However, metabolism could also be made a function of temperature.

### Thermal damage to tissue

An Arrhenius rate constant relationship is widely used to estimate cumulative thermal damage associated with burns of tissue, including skin [55-60]. This is equivalent to describing the conversion of a native form molecule to a denatured form by overcoming an energy barrier [61]. The Arrhenius rate constant-based expression for accumulation of irreversible thermal damage describes the process in terms of a rate at which the native form of a molecule moves to a transition state atop the energy barrier and then a final, denatured state. This simple description assumes that a single damage process, with Ω a dimensionless indicator of accrued tissue damage [56,62,63]:



where *A *= 2.9 × 10^37 ^s^-1 ^is the attempt rate, Δ*E *= 2.4 × 10^5 ^J mol^-1 ^is the effective activation energy, ℜ = 8.31 J mol^-1 ^K^-1 ^is the universal gas constant, and *T*(*z*, *t*) is the absolute temperature at a given location (here depth).

According to Lee and co-workers [57,58], the approximate threshold for the onset of thermal damage is 42°C. We therefore estimate the accumulated thermal damage using



where *z *is the depth into the tissue and *t*_*exp *_is the duration of the exposure. The cumulative damage index, Ω, has been related to tissue damage but can also be interpreted as the fraction of hypothetical indicator molecules that are denatured. Complete epidermal necrosis corresponds to Ω = 1. Although the heat induced damage to skin involves many processes, Eq. 7 is a simple model with zeroth-order kinetics [61].

### Initial and boundary conditions

#### Surface heating

The temperature of the surface node is elevated to the indicated temperature at t = 0 for a specified duration. The temperature of the core node deep in the skin is held constant at the ambient temperature of 37°C. In the 2-D case, all the lattice nodes at the skin surface are elevated to the indicated initial temperature at t = 0.

#### Spatially distributed heating

The far (left) boundary of the air layer away from the skin is held at 25°C while the core temperature is fixed at 37°C. The thermal current sources with different values is a function of z (Eq. 2), representing power deposition at different nodes, are turned on at t = 0 for a specified duration. This accounts for the spatially distributed power dissipation.

### Transport lattice solution

The transport lattice method employs locally interacting functional models to describe heat conduction, heat sources, removal of heat by perfusion and heat storage that are solved by Kirchhoff's Laws. We use electrical circuits which are mathematically analogous to the thermal processes (Fig. 1). The resulting electrical circuits were solved by Kirchhoff's laws using Berkeley SPICE version 3f5 [43,47], yielding currents and voltages of lattice elements. The voltages are converted to equivalent temperatures and displayed as temperature plots and images using Matlab (MathWorks, Natick, MA). A Pentium based computer (2 GHz CPU, 4 GB RAM) was used to obtain the solutions.

## Results

We demonstrate the use of a transport lattice approach to solve bioheat problems, using surface contact heating and spatially distributed heating of skin as illustrations.

### Method Validation

The transport lattice approach is validated theoretically by comparison to an one-dimensional analytical solution of the perfusion equation for a single medium with a homogeneous sink (equivalent to uniform perfusion). In this validation case, the surface of the medium was instantaneously changed to T_1 _(= 45°C) while the core was maintained at T_2 _(= 37°C). The initial condition assumed that the tissue temperature throughout was 37°C. The steady-state analytical solution to the bioheat equation (Eq. 1) for these conditions is the same as a spatially distributed uniform sink given by the equation [27]



Here *L *is the length of the model geometry (= 10 cm), *ρ*_*b *_is the density of blood, *c*_*b *_is the specific heat of blood, *ω*_*m *_is the perfusion rate and *k *is the thermal conductivity of the tissue.

**Figure 2:**
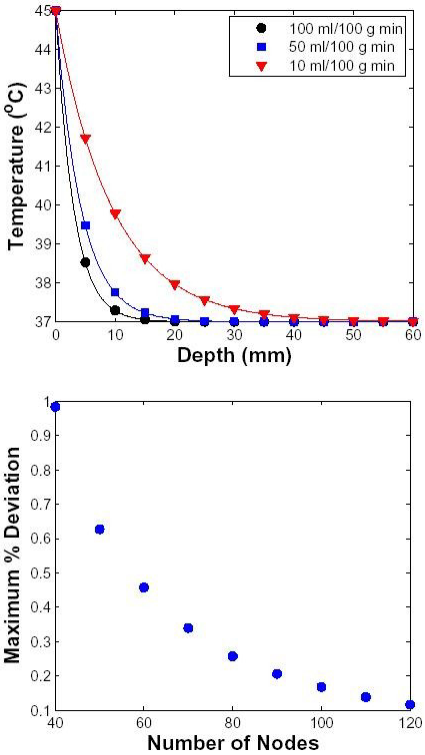
**Validation using one-dimensional geometry**. The validation model is a homogeneous section of a material (here dermis) with uniform perfusion (homogeneous sink), 10 cm in length and 100 *μm *× 100 *μm *in area. The surface of the tissue was elevated to 45°C at t = 0 s. The perfusion level (in ml/100 g min) was varied as shown in inset. The solid line represents the analytical solution (Eq. 8) and the symbols represent the transport lattice solution. Top: Steady-state temperature as a function of depth. The 10 cm long tissue is represented by 100 lattice elements, but the temperature profile is shown only to the depth of 6 cm. Bottom: The 10 cm long tissue is now represented by different number of lattice elements. Maximum % deviation as a function of the number of nodes used to represent the tissue is shown. The deviation is computed as the maximum discrepancy between the simulated temperature and the corresponding analytical value normalized to the step increase in temperature (here = 8°C).

The analytical result of Eq. 8 is compared with solutions of a one-dimensional transport lattice model with dermis tissue values assigned to the local models. For validation comparisons, the tissue (10 cm in length) is represented by a lattice with different number of nodes. The transport lattice temperature profiles agree remarkably well with Eq. 8 for different perfusion levels (Fig. 2). The performance of the transport lattice method was quantified by the maximum deviation of the transport lattice temperature profile from the analytical result normalized by (*T*_1_-37). As seen in Fig. 2, the numerical error is less than 1% when the geometry is represnted by only 40 nodes and becomes progressively better as more nodes are used.

### Surface Contact Heating – Transcutaneous Application

In this case, the skin surface temperature is approximated as increasing instantaneously from 33°C to 45°C at t = 100 s. This situation is encountered in transcutaneous blood gas sensing and, for more extreme heating, in skin burns due to a hot metal object and flash fire exposure.

#### Temporal distribution

**Figure 3:**
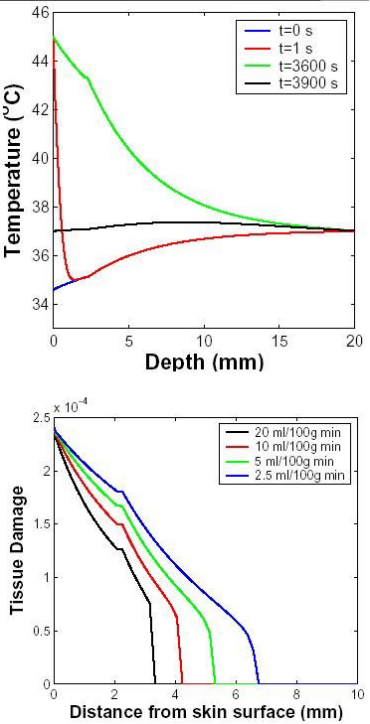
**Temperature distribution with surface contact heating**. The surface of the skin was elevated from 33°C to 45°C at t = 0 s for 3600 s. This approximates contact heating in which a metal (large thermal diffusivity) -encased heater with controlled temperature is held against the skin. Top: the temperature distribution as a function of skin depth with 10 ml/100 g min perfusion is shown for four different time points (inset in s). The four curves show the temperature profile before the application, immediately after the application, before the removal, and after the removal of the surface heating. Bottom: Tissue damage indicator predicted for the transcutaneous heating for four different perfusion levels (inset in ml/100 g min).

The spatial distribution of temperature and the resulting tissue damage from surface contact heating is shown in Fig. 3. The steady-state temperature distribution shows an exponential fall off with spatial decay constants dependent on the thermal properties of different layers of skin. Accumulated tissue damage is shown for different perfusion levels in Fig. 3. As expected intuitively, when the surface contact temperature is elevated, outer layers experience more damage than deeper regions of skin. For typical transcutaneous blood gas sensing conditions the estimated damage is small, even with prolonged skin contact to a 45°C surface.

#### Temperature-dependent perfusion

**Figure 4:**
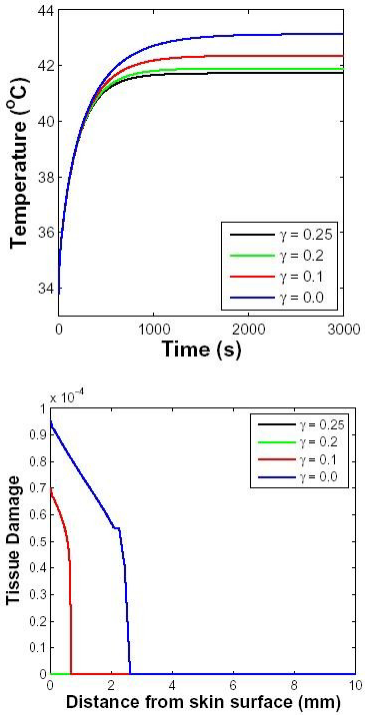
**Temperature-dependent perfusion distributions for contact heating**. The surface of the skin was elevated to 45°C at t = 100 s for 1 h. The perfusion level was dependent on local temperature with a temperature coefficient shown in inset. The basal perfusion rate was 10 ml/100 g min. Top: Temperature of skin close to surface as a function of time. Bottom: Tissue damage as a function of depth integrated over time (only the damage for two smallest perfusion values are discernible, hence the curves for higher perfusion rates are not seen in the figure).

Experiments have shown that heat stress causes a temperature-dependent response of the vasculature in tissues [64]. The blood flow in skin and muscle increases significantly for temperatures up to 43°C. Here temperature dependent blood perfusion in dermis and subcutaneous tissue is represented by *ω*_0_(1 + *γT*) where *ω*_0 _is the baseline perfusion and *γ *is the linear coefficient of temperature dependence. Figure 4 shows the temporal distribution of temperature close to skin surface for different values of *γ *for 1-hr exposure. As expected, increased perfusion causes a decline in local temperature. The accumulated tissue damage (Fig. 4) is also lower if the blood perfusion has a higher temperature coefficient, because the temperature rise is constrained.

### Spatially Distributed Heating by Microwave Exposure

The spatially distributed heating case illustrated here relates to heat generation (power dissipation) decaying exponentially with the distance within each skin tissue layer. We analyzed the case of an exposure to 10 GHz microwave for 3 s duration (a short-duration and high power microwave [HPM] exposure [21]).

#### Applied power level

**Figure 5:**
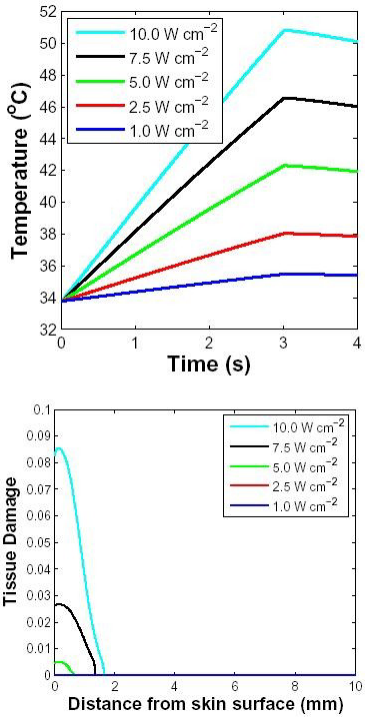
**Effect of applied power level for spatially distributed heating**. Response to a 10 GHz microwave pulse of 3 s duration with four different power densities (inset in W cm^-2^). The layer of air farthest from the skin (2 mm) was at 25°C, the skin surface was at 34°C before the pulse was applied and the core temperature was fixed at 37°C. The blood perfusion level was assumed to be 10 ml/100 g min. Top: Surface temperature as a function of time. Bottom: Tissue damage indicator, Ω, as a function of depth. Only the two highest levels of power generate noteworthy values of Ω (the plots for lower power levels are, therefore, not visible in the figure).

Figure 5 shows the change in skin surface temperature over time for different incident power levels. The skin is exposed to a 1 to 10 W cm^-2 ^10 GHz pulse for 3 s. The layer of air farthest from the skin was set at 25°C and the core (2 cm below the surface) was set to 37°C. This resulted in the skin/air interface having a steady-state temperature of 34°C before the microwave exposure. The skin/air interface has a power transmission coefficient (|*T*_*sa*_|^2^*Re*{*Z*_*a*_/*Z*_*e*_}) of 0.49 at 10 GHz. Applying 10 GHz microwave results in an essentially linear rise in temperature, in agreement with prediction using other methods. When the input power level is less than 5 W cm^-2^, the peak surface temperature is less than 42°C. When the microwave exposure is turned off, relaxation of the skin temperature occurs over a time scale of several seconds. Onset of tissue damage occurs when the local tissue temperature reaches 42°C. The distribution of tissue damage with depth is shown for different power densities (Fig. 5). Even for an incident power density as high as 10 W cm^-2^, the accumulated tissue damage for a 3 s exposure is far less than 1, even in the epidermis and dermis. Because of the large difference in the conductivity and permittivity of the dermis and subcutaneous fat, over 20% of the power deposited at the interface is reflected back into the dermis resulting in reduced power deposition in the fat layer. For power densities 5 W cm^-2 ^and less, the tissue temperature remains less than 42°C and the tissue damage indicator is negligible throughout the skin. This suggests that the tissue suffers no damage during this exposure.

#### Perfusion level

**Figure 6:**
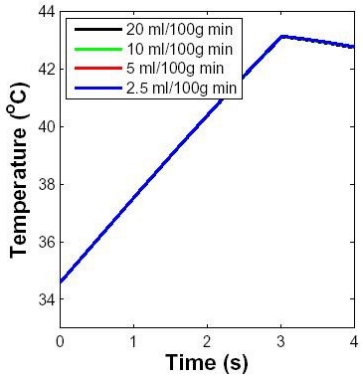
**Effect of blood perfusion level**. A 10 GHz microwave pulse of 3 s duration with power density of 5 W cm^-2 ^was considered for illustrative purposes. Blood perfusion levels in units of ml/100 g min are shown in inset. Skin surface temperature as a function of time is shown for these different perfusion levels. The different plots essentially overlap, showing that blood perfusion has negligible effect on temperature distribution in the case of a 3-sec 10 GHz exposure.

The peak surface temperature is shown in Fig. 6 for different blood flow rates. The basal perfusion levels in dermis and subcutaneous tissue were varied from 2.5 ml/100 g min to 20 ml/100 g min. The surface temperature distribution was nearly identical for this range of blood flow rate, a level of 20 ml/l00 g min is already at the high end of physiologic range for skin. This is consistent with the same skin temperature increases at different blood flow rates at 100 GHz reported by Nelson *et al. *[21].

#### Temperature distribution dynamics

**Figure 7:**
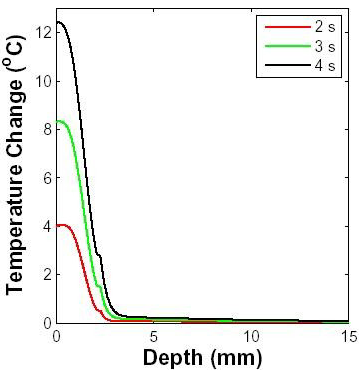
**Temperature distribution dynamics**. A 10 GHz microwave pulse of 3 s duration with a power density of 5 W cm^-2 ^was applied at t = 1 s. The layer of air farthest from the skin (2 mm) was set to 25°C, the skin surface was at 34°C before the RF field was applied and the core temperature (here 20 mm deep) was at 37°C. The temperature-independent blood perfusion level was assumed to be 10 ml/100 g min. Temperature change from baseline as a function of distance from skin surface is shown for different time points (2, 3 and 4 s).

Change in the spatial temperature distribution over time due to a 10 GHz pulse is shown in Fig. 7. The temperature of the outer layers of skin is below the core temperature of 37°C before the microwave exposure. During the pulse, the temperature of epidermis and dermis layers increases rapidly compared to deeper subcutaneous tissue. The temperature in the subcutaneous fat layer does not increase appreciably from its initial temperature because only a fraction of the incident power is transmitted into this region of the skin, and although heat absorbed in outer layers is removed partially by conduction, heat in the outer layers is mainly intercepted and removed by perfusion.

#### Skin heterogeneity

**Figure 8:**
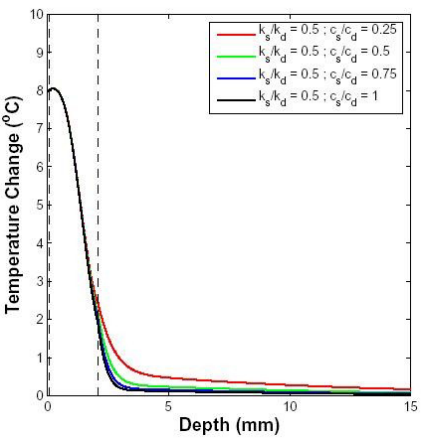
**Effect of skin layer parameters on temperature distribution for 10 GHz exposure**. Temperature change due to a 10 GHz pulse of 3 s duration with an incident power density of 5 W cm^-2^. The layer of air farthest from the skin was at 25°C, the skin surface was at 34°C before this RF field was applied and the core temperature was at 37°C. The blood perfusion level was assumed to be 10 ml/100 g min. The thermal conductivity and specific heat of dermis and subcutaneous tissue were varied relative to each other as shown in the inset.

The local elevated temperature at the interface of dermis and subcutaneous tissue observed in the spatial distribution of temperature during a 10 GHz exposure is due to different thermal properties of the homogeneous slabs that comprise the model. The effect of skin heterogeneity on temperature distribution is shown in Fig. 8. The specific heat and thermal conductivity of epidermis and subcutaneous tissue were varied relative to each other using a range of published values. In agreement with qualitative expectations, the temperature distribution prior to the end of microwave pulse shows that the larger the difference between the specific heat of the two layers, the larger the magnitude of the locally elevated temperature. However, most of the temperature increase is confined to the epidermis and dermis, as most of the incident power is deposited in those layers. It is expected that at higher frequencies, the temperature distribution in subcutaneous layers will be uniform because most of the RF energy will be deposited closer to the surface of the skin as the penetration depth diminishes.

### Two-dimensional temperature distribution

**Figure 9:**
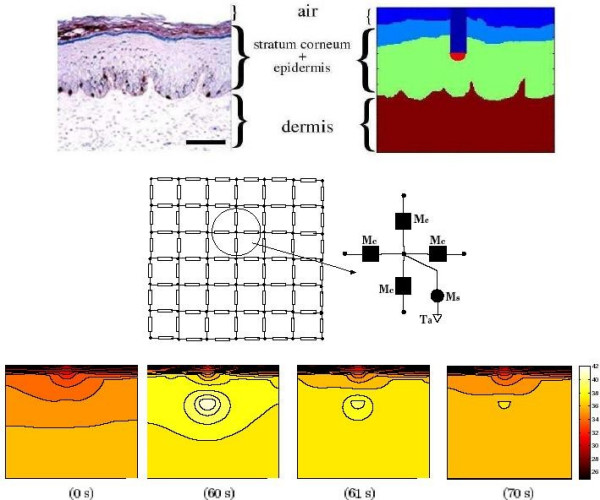
**Two-dimensional distribution of tissue temperature**. Top Left: Image of skin cross-section used in generating the simulation geometry. Scale bar: 50 *μm*. (Top Right). The Cartesian grid (ℓ = 1 *μm*) is superimposed on the model geometry to create the simulation geometry. Stratum corneum was assigned the same thermal properties as the epidermis. The hot wire is seen as dark blue region with a red tip. Center: A part of the 216 × 188 lattice is shown. At each node, five functional models are connected, four conduction models (M_*c*_) to the neighboring nodes and a fifth model (M_*s*_) representing heat storage and perfusion-transported heat to a reference temperature, *T*_*a*_. Depending on the type of underlying tissue (or air), the transport model at any node is one of four models shown in Fig. 1. The tip of the hot wire was elevated to 45°C at t = 10 s for 50 s with a rise and fall time of 5 s. Bottom Panels: Temperature contours at different time points: before the hot wire tip temperature is raised; just before the hot wire is turned off; immediately after the hot wire is turned off; 10 s later.

The use of transport lattice approach for predicting heat transport in spatially heterogeneous structures is further illustrated with a two-dimensional model of the skin. The model is derived from an image of a histological section of skin (Fig. 9). The temperature distribution from a thermally insulated wire (20 *μ*m diameter, 60 *μ*m length) with a hot tip that is inserted into the epidermis is also modeled. The model assumes that the tip of the metal wire (*k*_*w *_= 200 W m-^1^°C^-1^; *ρ*_*w *_= 8900 kg m^-3^; *c*_*w *_= 383 J kg-^1^°C^-1^) is enclosed in a thermally insulating material (*k*_*p *_= 0.15 W m^-1^°C^-1^; *ρ*_*p *_= 1200 kg m^-3^; *c*_*p *_= 2010 J kg^-1^°C^-1^). The skin model contains stratum corneum, epidermis and dermis. As before, the core temperature (37°C) is fixed at 2 cm from the skin surface by extending the subcutaneous layer. Before heating the wire conducts heat outwardly to the air, consistent with the isotherms. The temperature at the hot wire tip is increased to 45°C at t = 10 s. The temperature contours at different time points is shown in Fig. 9. The heterogeneity in skin structure is seen in the temperature contours immediately after the tip is heated, but then diminishes with time because the thermal properties of different skin regions differ only slightly. As intuitively expected, the thermal contours show a temperature gradient into the surrounding air when the wire tip is heated.

## Discussion

### Temperature-dependent perfusion

Both *in vivo *and *in vitro *studies have shown that the tissue response to heat stress is strongly temperature-dependent [33,64,65]. When heated to 41–43°C, temperatures that are commonly used in clinical hyperther-mia, the blood flow in normal tissues increases significantly [35]. In order to demonstrate the use of transport lattice approach to model temperature-dependent perfusion, we considered the simplest case of perfusion varying linearly with temperature. The perfusion was assumed to include a temperature-independent basal component and a temperature-dependent vasodilatory component.

As shown in Fig. 4, increased perfusion resulting from temperature dependence results in a lower peak temperature close to the skin surface. An increase in perfusion causes greater heat loss from the tissue into the blood, thus reducing the peak temperature. In addition, the temperature decays faster for a larger temperature coefficient after the removal of external heat source. The coefficient of temperature dependence could also utilize a non-linear function of temperature in the transport lattice method. This could reflect a decrease in perfusion at temperatures over 45°C resulting from heat-induced damage to blood capillaries.

A more comprehensive non-linear temperature dependent perfusion model has been applied in modeling hyperthermia. Tompkins *et al. *[66] used temperature-dependent models to show that blood perfusions initially increase with tissue temperature and then decrease at higher temperatures. Erdmann *et al. *[35] employed a Gaussian profile for temperature-dependence of perfusion increase between 37°C and 45°C, and a plateau for temperatures above 45°C. Our simpler linear dependence of perfusion on temperature is intended to demonstrate the use of a transport lattice method for heat transport in skin.

### Skin heterogeneity

We present a modular approach to modeling in which the skin is represented by three homogeneous layers, each with many interconnected local, steady state models that account for the local heat storage (heat capacity), local heat dissipation (local microwave heating) and local transport by both conduction and perfusion (Fig. 1). The existence of different thermal properties in adjacent layers of the model is particularly important for spatially distributed heat sources that extend through skin layers. In the case of a 10 GHz microwave radiation, the penetration depth is approximately 3 mm. Therefore, an exposure to 10 GHz radiation will cause a non-uniform temperature distribution within the skin.

As shown in Fig. 8, differences in thermal conductivity and specific heat of different layers of the skin create different temperature profiles, especially in the subcutaneous fat layer. However, with higher frequency RF radiation, the penetration depth decreases, and the difference in temperature profiles in the skin will diminish.

### Tissue damage

Prolonged exposure to elevated temperatures can cause tissue damage by, for example, protein alteration or denaturation, often followed by recognizable changes in the optical properties of tissue [67]. The rate of the transition from natural to denatured states is governed approximately by the Arrhenius rate equation (Eq. 7). The lipid bilayer components of the cells are most vulnerable to thermal damage because they are held together only by forces of hydration [68]. Exposure to ambient microwave fields has been shown to cause tissue damage. The rate of tissue heating has a large dependence on the density of dipoles, resulting in a much slower microwave heating in fatty tissues [69].

When skin is exposed to a 10 GHz pulse of 3 s duration, the tissue damage indicator near the skin surface may be as high as 0.08, which suggests some damage at high power levels (Fig. 5). This exposure generates a surface temperature of approximately 51°C. Human pain perception studies have shown that the threshold for perception corresponds to a significantly lower mean skin temperature of 44°C [22]. Thus, a relatively non-damaging exposure might cause significant pain.

## Conclusions

Transport of heat by conduction, and by temperature-dependent, spatially heterogeneous blood perfusion, is predicted using a transport lattice model. This approach uses interconnected, local, steady state models for transport and storage, to together represent the Pennes bioheat equation. The thermal system model of the skin was solved by exploiting the mathematical analogy between local thermal models and local electrical (charge transport) models, thereby allowing robust, circuit simulation software to obtain solutions to Kirchhoff's laws for the system model. The skin model has a nonperfused viable epidermis, and deeper regions of dermis and subcutaneous tissue with perfusion that was constant or temperature-dependent. Spatially distributed heating and surface heating cases were considered. Accumulated thermal damage was estimated by using an Arrhenius type relation at locations where viable tissue temperature exceeds 42°C. Prediction of spatial temperature distributions was also illustrated with a two-dimensional model of skin created from an image. Validation of the transport lattice approach using experimental data is necessary for practical application of this method.

## Authors' Contributions

TRG constructed and solved the several transport lattice models and wrote much of the manuscript. DAS computed the reflected and transmitted power in the skin layers, contributed to construction and solution of the models, and to writing of the manuscript. GTM provided guidance and advice with respect to thermal modeling, and helped write the manuscript. JCW conceived the local transport lattice model for solving the bioheat equation, provided overall guidance and helped with interpretation of results and writing the manuscript. All authors read the final manuscript.

**Table 2:** Definition of model parameters used in the transport lattice simulations. The parameter values were obtained from the sources cited in the rightmost column.

Air		
*R*_*ca*_	conduction model	= (*k*_*a*_ℓ)^-1^
*C*_*a*_	storage model	= *ρc*_*a*_ℓ^3^

Epidermis		

*R*_*ce*_	conduction model	= (*k*_*e*_ℓ)^-1^
*C*_*e*_	storage model	= *ρc*_*e*_ℓ^3^
*I*_*e*_	distributed local power	= *P*(*z*, *t*)ℓ^3^

Dermis		

*R*_*cd*_	conduction model	= (*k*_*d*_ℓ)^-1^
*C*_*d*_	storage model	= *ρc*_*d*_ℓ^3^
*R*_*pd*_	conduction model	= ℓ^3^*ω*_*m*_*c*_*b*_*ρ*_*b*_
*I*_*d*_	distributed local power	= *P*(*z*, *t*)ℓ^3^

Subcutaneous Tissue		

*R*_*cs*_	conduction model	= (*k*_*s*_ℓ)^-1^
*C*_*s*_	storage model	= *ρc*_*s*_ℓ^3^
*R*_*ps*_	conduction model	= ℓ^3^*ω*_*m*_*c*_*b*_*ρ*_*b*_
*I*_*s*_	distributed local power	= *P*(*z*, *t*)ℓ^3^
